# ﻿Two new species of Hypodontolaiminae (Nematoda, Chromadorida, Chromadoridae) from the Yellow Sea with a phylogenetic analysis in the subfamily

**DOI:** 10.3897/zookeys.1190.113418

**Published:** 2024-01-30

**Authors:** Huixin Liang, Wen Guo, Chunming Wang

**Affiliations:** 1 College of Life Sciences, Liaocheng University, Liaocheng, 252059, China Liaocheng University Liaocheng China

**Keywords:** China, *Dichromadora*, marine nematode, *Neochromadora*

## Abstract

Two new species of Hypodontolaiminae, *Dichromadoramedia***sp. nov.** and *Neochromadoraparabilineata***sp. nov.**, were isolated and described from the Yellow Sea, China. *Dichromadoramedia***sp. nov.** is characterized by four long cephalic setae, the amphidial fovea transverse oval in the male and slit-shaped in the female, the pharynx with a single posterior bulb, spicules curved and distally bifurcated, gubernaculum jointed, four (1+3) precloacal supplements papilliform, and the tail conical elongated with a short spinneret. *Neochromadoraparabilineata***sp. nov.** is characterized by the buccal cavity with one large hollow dorsal tooth and two small subventral teeth, the pharynx with an obvious posterior bulb, spicules L-shaped and widened medially, gubernaculum boat-shaped, seven cup-shaped and equidistant precloacal supplements, and a long and gradually tapering tail. The phylogenetic analysis of maximum likelihood and Bayesian inference based on rDNA sequences confirmed the taxonomic positions of *Neochromadoraparabilineata***sp. nov.** and *Dichromadoramedia***sp. nov.** within Hypodontolaiminae. Tree topology in Hypodontolaiminae shows the genera *Neochromadora*, *Dichromadora*, *Ptycholaimellus*, and *Spilophorella* as polyphyletic groups, and the genus *Chromadorita* as a paraphyletic group.

## ﻿Introduction

Nematodes are the most widely distributed and diverse metazoans on the planet, and a large number of nematode species still remain unidentified (Hodda 2022).Chromadoridae Filipjev, 1917 is one of the largest families of nematodes and shown as a monophyletic group with synapomorphies of male monorchid with an anterior testis; precloacal supplements cup-shaped or absent but never tubular; females with two reflexed ovaries, anterior to the right of the intestine, posterior to the left of the intestine (Tchesunov 2014). Chromadoridae has been reviewed systematically by Wieser (1954a), Gerlach and Riemann (1973/1974), Lorenzen (1981, 1994), Tchesunov (2014), and Venekey et al. (2019), and its phylogenetic relationships have been analyzed based on rDNA sequences by Holterman et al. (2008), Leduc et al. (2017), Venekey et al. (2019), and Guo et al. (2023). The number of rDNA sequences in Chromadoridae have rapidly increased in GenBank: up to now, 250 sequences from 20 genera of Small Subunit (SSU) and 145 sequences from 11 genera of D2–D3 fragment of Large Subunit (LSU) have been deposited in GenBank. In our study of marine nematode taxonomy from the Rizhao coast, at the Yellow China Sea, two new species from the subfamily Hypodontolaiminae are described, *Dichromadoramedia* sp. nov. and *Neochromadoraparabilineata* sp. nov. Ribosome DNA sequences from these two new species and three other species, *Dichromadorasinica* Huang & Zhang, 2010, *Dichromadoramajor* Huang & Zhang, 2010, and *Dichromadoramultisetosa* Huang & Zhang, 2010, are acquired for phylogenetic analysis.

## ﻿Materials and methods

### ﻿Sample collection

In July 2022, undisturbed samples were collected from intertidal sediments in the Rizhao coast, the Yellow China Sea. Sediments samples were vertically collected with a syringe (2.6 cm internal diameter) to a depth of 8 cm and subdivided into 0–2 and 2–8 cm depth parts. Sediments used for morphological analysis were fixed in a 10% formalin solution in seawater and for molecular analysis were preserved in 95% ethanol. Formalin-fixed samples were stained with 0.1% of Rose Bengal for more than 24 hours. Meiofauna were extracted from the sediment through Ludox centrifugation (Higgins and Thiel 1988), washed through two sieves with mesh sizes of 500 μm and 45 μm with tap water to separate meiofauna from macrofauna (larger than 500 μm), transferred to a grid-lined Petri dish, and sorted under a stereoscopic microscope. Nematodes were transferred into a mixture of ethanol (50%) and glycerin in the ratio 1:9 by volume with the ethanol slowly evaporated away (McIntyre and Warwick 1984). Nematodes were mounted in glycerin on permanent slides. Descriptions were made using an Axiscope–5 differential interference contrast microscope (Zeiss, Germany). Line drawings were made with the aid of iPad (Apple, USA), and photographs were taken with the aid of ZEN software (Zeiss). Type specimens were deposited in the Institute of Oceanology, Chinese Academy of Sciences, Qingdao.

Sediments used for molecular analysis were washed and separated as with formalin-fixed samples except without Rose Bengal dying. Seven male specimens of *D.media* sp. nov., two male specimens of *N.parabilineata* sp. nov., five male specimens of *D.sinica*, two male specimens of *D.major*, and two male specimens of *D.multisetosa* were separated and confirmed on the temporary slides.

### ﻿DNA extraction, PCR amplification, and phylogenetic analysis

Genomic DNA was extracted with DNeasy Blood & Tissue kit (Qiagen, Germany) and used as amplification templates for nearly full length SSU rDNA gene, with primers of G18S4F (5’ – GCT TGT CTC AAA GAT TAA GCC – 3’) / 18PR (5’ – TGA TCC WMC RGC AGG TTC AC – 3’) (Blaxter et al. 1998), and D2–D3 fragment of LSU rDNA gene with primers of D2A (5’ – ACA AGT ACC GTG AGG GAA AGT TG – 3’) / D3B (5’ – TCG GAA GGA ACC AGC TAC TA – 3’) (Nunn 1992). PCR was conducted as described by Zhao et al. (2015). The PCR product was sequenced by Genewiz (China). The sequences were assembled in Genious v. 6.1.2. The newly obtained SSU rDNA sequences have the accession numbers as follows: *D.media* sp. nov. OR479913, *N.parabilineata* sp. nov. OR126985, *D.sinica*OR479916, *D.major*OR479911, and *D.multisetosa*OR479915; the D2–D3 fragment of LSU rDNA sequence accession numbers are *D.media* sp. nov. OR479918, *N.parabilineata* sp. nov. OR135360, *D.sinica*OR479914, *D.major*OR479912, and *D.multisetosa*OR479917. All have been deposited in GenBank.

Sequences of subfamily Hypodontolaiminae in GenBank were used for phylogenetic analysis. Forty-nine SSU rDNA sequences from seven genera (Table 1) longer than 600 bp were selected and aligned with the Muscle algorithm. Substitution models of (GTR (general time-reversible) + G (gamma distribution) + I (proportion of invariable sites)) were selected as the best-fit model and the analysis was rooted with *Latronemawhataitai* Leduc & Zhao, 2015 (accession number KR048680). Sixteen D2–D3 fragment of LSU rDNA sequences from five genera (Table 2) were selected and aligned with the Muscle algorithm. Substitution models of (GTR (general time-reversible) + G (gamma distribution)) were selected as the best-fit model, and the analysis was rooted with *Latronemawhataitai* (accession number KR04868).

**Table 1.** SSU information of samples used for phylogenetic analysis.

Species	GenBank number	Reference	Locality
Chromadoritaaff.leuckarti	MF409784.1	Schenk et al. 2018	Germany
Chromadoritacf.leuckarti	FJ040473.1	Holterman et al. 2008	–
*Chromadoritahumila*	OQ396742.1	Sun et al. 2023	China
*Chromadoritaleuckarti*	FJ969119.1	van Megen et al. 2009	–
*Chromadoritaleuckarti*	KJ636254.1	Bert et al. 2014	–
*Chromadoritaspinicauda*	OK317201.1	Leduc and Zhao 2023	New Zealand
*Dichromadoramajor*	OR479911.1	Wang et al. 2023	China
*Dichromadoramedia* sp. nov.	OR479913.1	Wang et al. 2023	China
*Dichromadoramultisetosa*	OR479915.1	Wang et al. 2023	China
*Dichromadorasimplex*	MG669747.1	Macheriotou et al. 2018	Vietnam
*Dichromadorasinica*	OR479916.1	Wang et al. 2023	China
*Dichromadora* sp.	MN250081.1	Pereira et al. 2019	Beaufort Sea (USA)
*Dichromadora* sp.	FJ040506.1	Holterman et al. 2008	–
*Dichromadora* sp.	MN250085.1	Pereira et al. 2019	Beaufort Sea (USA)
*Dichromadora* sp.	MG669748.1	Macheriotou et al. 2018	Netherlands
*Dichromadora* sp.	MN250044.1	Pereira et al. 2019	Beaufort Sea (USA)
*Dichromadora* sp.	MK626828.1	Tytgat et al. 2019	Vietnam
*Dichromadora* sp.	MG669752.1	Macheriotou et al. 2018	Vietnam
*Dichromadora* sp.	MG669751.1	Macheriotou et al. 2018	Vietnam
cf. *Dichromadora* sp.	KJ636253.1	Bert et al. 2014	–
*Hypodontolaimusinaequalis*	MG669813.1	Macheriotou 2018	Netherlands
*Hypodontolaimusinaequalis*	MG669812.1	Macheriotou et al. 2018	Netherlands
*Innocuonematentabundum*	AY854208.1	Meldal 2005	Southampton (United Kingdom)
*Innocuonematentabundum*	JN968213.1	Fonseca et al. 2012	–
*Neochromadorabilineata*	OQ396744.1	Sun et al. 2023	China
*Neochromadoraparabilineata* sp. nov.	OR126985.1	Wang et al. 2023	China
*Neochromadorapoecilosomoides*	OQ396720.1	Chu et al. 2023	China
*Neochromadora* sp.	AY854210.1	Meldal 2005	Southampton (United Kingdom)
*Neochromadora* sp.	MG669893.1	Macheriotou et al. 2018	Netherlands
*Neochromadora* sp.	KX944147.1	Avo et al. 2017	Mira estuary (Portugal)
*Neochromadora* sp.	MN250121.1	Pereira et al. 2019	Beaufort Sea (USA)
*Neochromadora* sp.	JN968279.1	Fonseca et al. 2012	–
*Ptycholaimellusareniculus*	MG669987.1	Macheriotou et al. 2018	Vietnam
*Ptycholaimellusareniculus*	MG669988.1	Macheriotou et al. 2018	Vietnam
*Ptycholaimellusbrevisetosus*	MK626833.1	Tytgat et al. 2019	Vietnam
*Ptycholaimellusbrevisetosus*	MK626834.1	Tytgat et al. 2019	Vietnam
*Ptycholaimellusbrevisetosus*	MK626809.1	Tytgat et al. 2019	Vietnam
*Ptycholaimellusbrevisetosus*	MG669989.1	Macheriotou et al. 2018	Vietnam
*Ptycholaimellusocellatus*	OQ538290.1	Sun et al. 2023	China
*Ptycholaimellusspiculuncus*	OK317202.1	Leduc and Zhao 2023	New Zealand
*Ptycholaimellus* sp.	FJ040472.1	Holterman et al. 2008	–
*Ptycholaimellus* sp.	KX944158.1	Avo et al. 2017	Mira estuary (Portugal)
*Ptycholaimellus* sp.	JN968285.1	Fonseca et al. 2012	–
*Ptycholaimellus* sp.	MG669992.1	Macheriotou et al. 2018	Vietnam
*Ptycholaimellus* sp.	JN968257.1	Fonseca et al. 2012	–
*Spilophorellaaberrans*	MG670031.1	Macheriotou et al. 2018	Vietnam
*Spilophorellaparadoxa*	AY854211.1	Meldal 2005	Southampton (United Kingdom)
*Spilophorellaparadoxa*	JN968274.1	Fonseca 2012	–
*Spilophorella* sp.	MG670032.1	Macheriotou et al. 2018	Vietnam
*Latronemawhataitai*	KR048680.1	Leduc and Zhao 2016	–

**Table 2.** D2–D3 fragment of LSU information of samples used for phylogenetic analysis.

Species	GenBank number	Reference	Locality
*Chromadoritahumila*	OQ396736.1	Sun et al. 2023	China
*Chromadoritaspinicauda*	OK317226.1	Leduc and Zhao 2023	New Zealand
*Dichromadoracucullata*	GU003894.1	Rodrigues et al. 2010	USA
*Dichromadoramajor*	OR479912.1	Wang et al. 2023	China
*Dichromadoramedia* sp. nov.	OR479918.1	Wang et al. 2023	China
*Dichromadoramultisetosa*	OR479917.1	Wang et al. 2023	China
*Dichromadorasinica*	OR479914.1	Wang et al. 2023	China
*Dichromadora* sp.	KC755220.1	Vogt et al. 2014	Wilhelmshaven (Germany)
Neochromadoraaff.poecilosoma	KC755218.1	Vogt et al. 2014	Jadebusen (Germany)
*Neochromadoraparabilineata* sp. nov.	OR135360.1	Wang et al. 2023	China
*Neochromadorapoecilosomoides*	OQ417520.1	Sun et al. 2023	China
*Neochromadora* sp.	KC755219.1	Vogt et al. 2014	Wilhelmshaven (Germany)
*Ptycholaimellusocellatus*	OQ466609.1	Sun et al. 2023	China
*Ptycholaimellusspiculuncus*	OK317227.1	Leduc and Zhao 2023	New Zealand
*Spilophorella* sp.	DQ077766.1	De Ley et al. 2009	Mexico
*Spilophorella* sp.	GU003892.1	Rodrigues et al. 2010	USA
*Latronemawhataitai*	KR048681.1	Leduc and Zhao 2016	New Zealand

The ML analyses were performed with Mega X with 1000 bootstrap replicates. The BI analyses were constructed with CIPRES (http://www.phylo.org/) and MrBayes on XSEDE v. 3.2.7a were used; the trees were run with chain length of 10,000,000, burn-in frac = 0.25. The topology of the resulting trees was visualized using FigTree v. 1.4.3 and refined with PowerPoint.

## ﻿Results

### ﻿Taxonomic account

**Order Chromadorida Chitwood, 1933**

**Family Chromadoridae Filipjev, 1917**

**Subfamily Hypodontolaiminae De Coninck, 1965**

#### ﻿Genus Dichromadora

Taxon classificationAnimaliaChromadoridaChromadoridae

Kreis, 1929

1E40A804-162B-502B-B8F3-0B51495776C0

##### Diagnosis

**(based on Venekey et al. 2019).** Cuticle with homogeneous ornamentation and a pronounced lateral differentiation of two longitudinal rows of enlarged dots. Six outer labial papillae and four cephalic setae in separate circles. Amphideal fovea transverse slit-like and loop shaped. Buccal cavity with a triangular hollow dorsal tooth or a large dorsal tooth and two additional ventrosublateral ones; denticles can be present. Peribuccal pharyngeal tissue not swollen anteriorly or with an asymmetrical dorsal swelling; a distinct posterior pharyngeal bulb. Precloacal supplements present or absent.

##### Remarks.

The genus *Dichromadora* was erected by Kreis in 1929 with the type species *Dichromadoramicrodonta* Kreis, 1929 with the genus characters the cuticle with two longitudinal rows of dots, small tooth, pharynx bulb big and round, ovaries paired symmetrical and reflexed, and males with or without precloacal supplements. Six species from the genus *Chromadora* (*C.cephalata* Steiner, 1916, *C.cricophana* Filipjev, 1922, *C.geophila* de Man, 1876, *C.parapoecilosoma* Micoletzky, 1922, *C.sabulicola* Filipjev, 1918, *C.setosa* Bütschli, 1874) were transferred to *Dichromadora* by Kreis (1929). Later, *D.hyalocheile* De Coninck & Schuurmans Stekhoven, 1933, *D.tobaensis* Schneider, 1937, *D.strandi* Allgén, 1940, *D.punctata* Schuurmans Stekhoven, 1950, *D.tenuicauda* Schuurmans Stekhoven, 1950, and *D.abnormis* Gerlach, 1953a were described. Wieser (1954a) described *D.dissipata* Wieser, 1954a, revised the genus characters based mainly on tooth shape and provided new combinations. After Wieser (1954a), *D.apapillata* Timm, 1961, *D.arcospiculum* Timm, 1961, *D.simplex* Timm, 1961, *D.islandica* Kreis, 1963, *D.scandula* Lorenzen, 1966, and *D.cucullata* Lorenzen, 1973 were described and Gerlach and Riemann (1973) presented a list of sixteen species. Later, 12 species (*D.amphidiscoides* Kito, 1981, *D.abyssalis* Bussau, 1993, *D.gathuai* Muthumbi & Vincx, 1998, *D.loiseae* Muthumbi & Vincx, 1998, *D.longicaudata* Muthumbi & Vincx, 1998, *D.quadripapillata* Muthumbi & Vincx, 1998, *D.parasimplex* Dashchenko, 2002, *D.parva* Vermeeren, Vanreusel & Vanhove, 2004, *D.polaris* Vermeeren, Vanreusel & Vanhove, 2004, *D.polarsternis* Vermeeren, Vanreusel & Vanhove, 2004, *D.southernis* Vermeeren, Vanreusel & Vanhove, 2004 and *D.weddellensis* Vermeeren, Vanreusel & Vanhove, 2004) were described. Huang and Zhang (2010) described three species from the China Sea, *D.major*, *D.multisetosa*, and *D.sinica*, and provided a short review of *Dichromadora*. *Dichromadoraabyssalis* was considered as valid by Holovachov (2020) based on the high quality descriptions and illustrations despite not following the International Code of Zoological Nomenclature (Venekey et al. 2019). With the addition of *D.rigida* Thanh, Tu & Gagarin, 2016 and *D.agilis* Long, Gagarin & Tu, 2022, 35 species are currently considered as valid (based on Venekey et al. 2019):

1. *Dichromadoraabnormis* Gerlach, 1953 (Italy, San Rossore and Tirrenia beaches)

2. *Dichromadoraabyssalis* Bussau, 1993 (SE Pacific, Peru Basin)

3. *Dichromadoraagilis* Long, Gagarin & Tu, 2022 (Vietnam, Quảng Ninh)

4. *Dichromadoraamphidiscoides* Kito, 1981 (Japan, Oshoro Bay)

5. *Dichromadoraantarctica* (Cobb, 1914) (Antarctica, Cape Royd; = *Spilophoraantarctica* Cobb, 1914)

6. *Dichromadoraapapillata* Timm, 1961 (Indian Ocean, Bay of Bengal)

7. *Dichromadoraarcospiculum* Timm, 1961 (Indian Ocean, Bay of Bengal)

8. *Dichromadoracephalata* (Steiner, 1916) (Arctic Ocean, Barents Sea; = *Chromadoracephalata* Steiner, 1916, *Chromadoracricophana* Filipjev, 1922)

9. *Dichromadoracucullata* Lorenzen, 1973 (North Sea, Baltic Sea, Helgoland)

10. *Dichromadoradissipata* Wieser, 1954 (Chile, Seno de Reloncaví)

11. *Dichromadoragathuai* Muthumbi & Vincx, 1998 (Indian Ocean, Kenyan coast)

12. *Dichromadorageophila* (de Man, 1876) (North Sea, Netherlands; = *Chromadoracanadensis* (Cobb, 1914), *Chromadorageophila* (de Man, 1876), *Hypodontolaimusgeophilus* (de Man, 1876), *Spilipherageophila* de Man, 1876, *Spilipheraspectabilis* Allgén, 1929)

13. *Dichromadoragracilis* (Kreis, 1929) (France, Trebeurden; = *Spilophorellagracilis* Kreis, 1929)

14. *Dichromadorahyalocheile* De Coninck & Schuurmans Stekhoven, 1933 (Belgium, Oostende)

15. *Dichromadoraislandica* Kreis, 1963 (Iceland, Eyjafjörður)

16. *Dichromadoraloiseae* Muthumbi & Vincx, 1998 (Indian Ocean, Kenyan coast)

17. *Dichromadoralongicaudata* Muthumbi & Vincx, 1998 (Indian Ocean, Kenyan coast)

18. *Dichromadoramajor* Huang & Zhang, 2010 (China, Yellow Sea)

19. *Dichromadoramedia* sp. nov. (China, Yellow Sea)

20. *Dichromadoramicrodonta* Kreis, 1929 (France, English Channel)

21. *Dichromadoramultisetosa* Huang & Zhang, 2010 (China, Yellow Sea)

22. *Dichromadoraparasimplex* Dashchenko, 2002 (New Guinea, Astrolabe Bay)

23. *Dichromadoraparva* Vermeeren, Vanreusel & Vanhove, 2004 (Antarctic Sea, Halley Bay)

24. *Dichromadorapolaris* Vermeeren, Vanreusel & Vanhove, 2004 (Antarctic Sea, Halley Bay)

25. *Dichromadorapolarsternis* Vermeeren, Vanreusel & Vanhove, 2004 (Antarctic Sea, Halley Bay)

26. *Dichromadorapunctata* Schuurmans Stekhoven, 1950 (Mediterranean, Villefranche Bay)

27. *Dichromadoraquadripapillata* Muthumbi & Vincx, 1998 (Indian Ocean, Kenyan coast)

28. *Dichromadorarigida* Thanh, Tu & Gagarin, 2016 (Vietnam, Yen River Estuary)

29. *Dichromadorascandula* Lorenzen, 1966 (North Sea, Schleswig-Holstein)

30. *Dichromadorasimplex* Timm, 1961 (Indian Ocean, Bay of Bengal)

31. *Dichromadorasinica* Huang & Zhang, 2010 (China, Yellow Sea)

32. *Dichromadorasouthernis* Vermeeren, Vanreusel & Vanhove, 2004 (Antarctic Sea, Halley Bay)

33. *Dichromadorastrandi* Allgén, 1940 (Norway, Knivskjaerodden)

34. *Dichromadoratobaensis* Schneider, 1937 (Indonesia, Sumatra)

35. *Dichromadoraweddellensis* Vermeeren, Vanreusel & Vanhove, 2004 (Antarctic Sea, Halley Bay)

#### ﻿ Dichromadora media sp. nov.

Taxon classificationAnimaliaChromadoridaChromadoridae

556D6323-23E9-5B4D-B82A-B051FC64DF45

https://zoobank.org/4F7D70E0-54B3-422C-9A87-F496EA14C572

**Figure 1.**
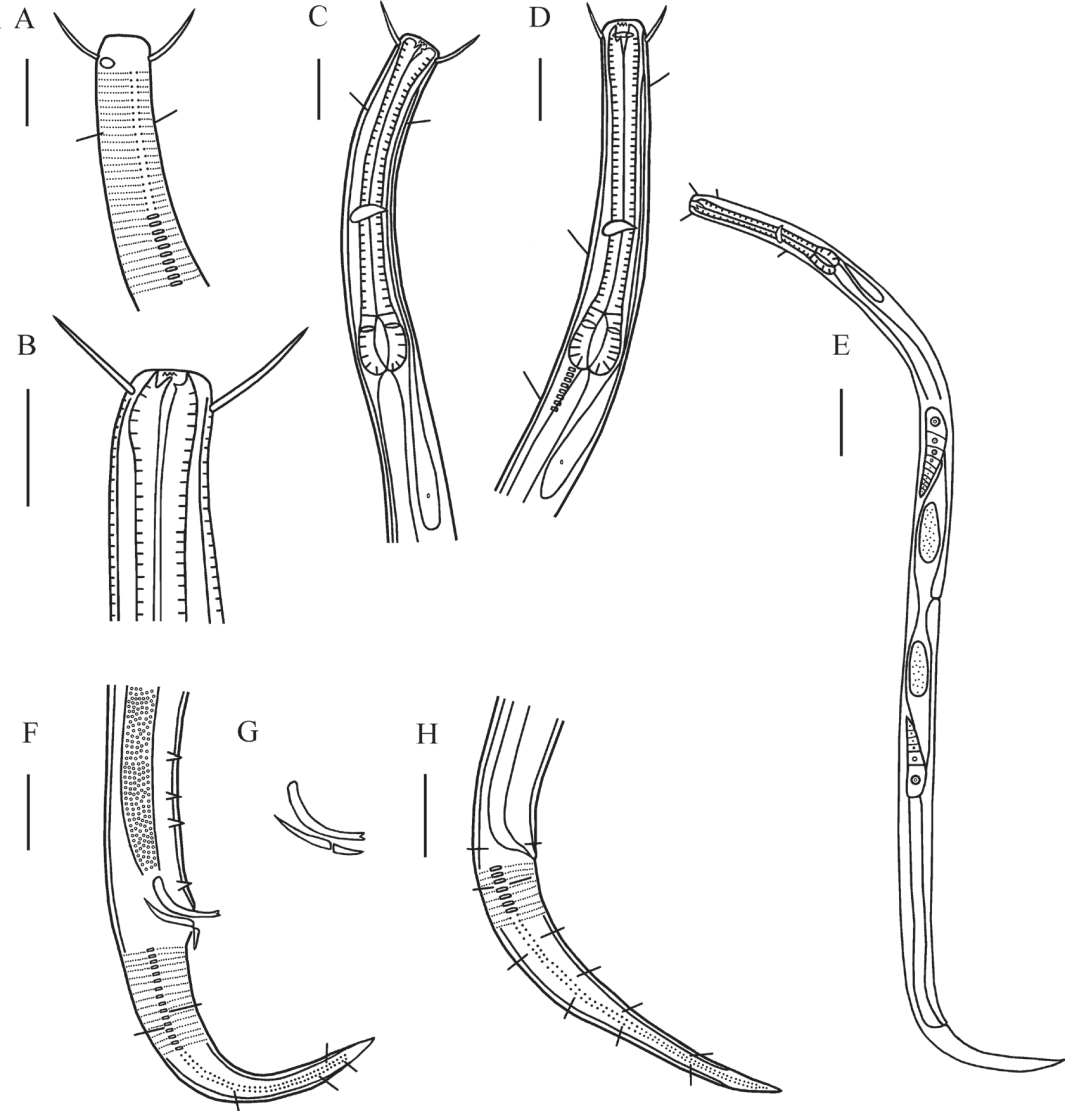
*Dichromadoramedia* sp. nov. **A** lateral view of male anterior region showing cuticle and amphidial fovea (holotype) **B** lateral view of male buccal cavity (holotype); **C** lateral view of male anterior region showing pharyngeal region (holotype) **D** lateral view of female anterior region showing buccal cavity, amphidial fovea and pharyngeal region (22ZJT8-2-7) **E** lateral view of female whole body (22ZJT8-2-7) **F** lateral view of male posterior body, showing precloacal supplements and tail (holotype); **G** lateral view of spicules and gubernaculum (22ZJT8-2-5) **H** lateral view of female posterior body showing tail (22ZJT8-2-7). Scale bars: 20 µm (**A–D, F, H**); 50 µm (**E**).

**Figure 2.**
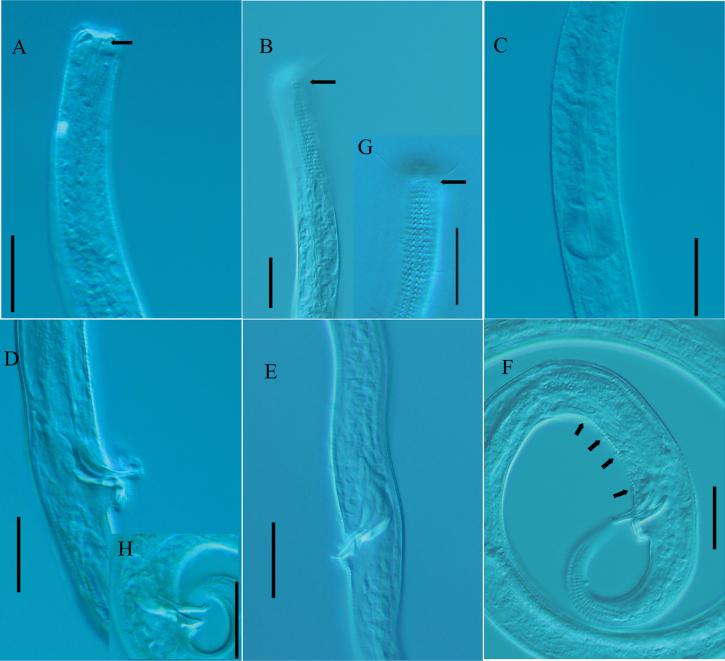
*Dichromadoramedia* sp. nov. **A** lateral view of male anterior region showing tooth (arrow) (holotype) **B** lateral view of male anterior region showing cuticle, amphidial fovea (arrow) (holotype) **C** lateral view of male anterior region showing pharyngeal region (holotype) **D** lateral view of male posterior body, showing spicules (holotype) **E** lateral view of male posterior body, showing gubernaculum (22ZJT8-1-2) **F** lateral view of male posterior body, showing precloacal supplements (arrows) (holotype) **G** lateral view of female anterior region showing amphidial fovea (arrow) (22ZJT8-1-2) **H** lateral view of distal end of spicules (22ZJT8-2-5). Scale bars: 20 µm.

Figs 1 , 2 , Table 3

##### Diagnosis.

Medium body size, cuticle with transverse rows of dots and a lateral differentiation of two longitudinal larger dots, four long cephalic setae, buccal cavity large with one large, hollow and straight dorsal tooth and two small ventrosublateral teeth, amphidial fovea transverse oval in male and slit-shaped in female, pharynx with single posterior bulb, spicules curved and distally bifurcated, gubernaculum jointed, four (1+3) papilliform precloacal supplements, tail conical elongated with short spinneret.

##### Material examined.

Four males and three females were measured and studied. ***Holotype***: ♂ 1 on slide 22ZJT8–1–2; ***Paratypes***: ♂ 2 on 22ZJT8–1–2, ♂ 3 on 22ZJT8–1–2, ♂ 4 on 22ZJT8–2–5, ♀ 1 on 22ZJT8–2–7, ♀ 2 on 22ZJT8–2–7, and ♀ 3 on 22ZJT8–2–6.

##### Type locality and habitat.

Rizhao coast, Shandong Province, China, 35°27′N, 119°35′E, 0–2 cm sediment depth, sandy sediment.

##### Measurements.

All measurement data are given in Table 3.

**Table 3.** Measurements of *Dichromadoramedia* sp. nov. (in µm except for ratios).

Characters	Holotype	Paratypes	Paratypes
	male	males (*n* = 3)	females (*n* = 3)
Total body length	925	895±14.5(881–910)	812±44.6(761–844)
Maximum body diameter	23	22.3±0.6(22–23)	27.7±0.6(27–28)
Head diameter	15	14.3±0.6(14–15)	16±0(16–16)
Length of cephalic setae	18	18.3±0.6(18–19)	10.7±0.6(10–11)
Amphidial fovea width	4	4±0(4–4)	8±0(8–8)
Amphidial fovea from anterior end	7	7.3±0.6(7–8)	4.7±0.6(4–5)
Body diameter at amphidial fovea	15	14.3±0.6(14–15)	16±0(16–16)
Nerve ring from anterior end	64	64.3±5.1(60–70)	64±4.6(59–68)
Body diameter at nerve ring	18	18±0(18–18)	20.3±1.2(19–21)
Pharynx length	116	114.7±2.5(112–117)	119±4.4(116–124)
Pharynx bulb length	22	22.3±0.6(22–23)	25.3±1.5(24–27)
Body diameter at base of pharynx	19	19±0(19–19)	22.3±1.2(21–23)
Cloacal/anal body diameter	23	22.3±0.6(22–23)	15.7±0.6(15–16)
Spicules length along arc	23	24.3±0.6(24–25)	–
Gubernaculum length	23	22±1(21–23)	–
Vulva from anterior end	–	–	399±16.1(381–412)
Body diameter at vulva	–	–	27.3±0.6(27–28)
V%	–	–	49.2±0.8(48.6–50.1)
Precloacal supplements	1+3	1+3	–
Tail length	103	107±3.5(105–111)	106.7±6.8(99–112)
a	40.2	40.1±1.2(38.9–41.4)	29.4±1.9(27.2–30.8)
b	8.0	7.8±0.1(7.8–7.9)	6.8±0.3(6.6–7.2)
c	9.0	8.4±0.4(7.9–8.7)	7.6±0.2(7.4–7.7)
c’	4.5	4.8±0.2(4.6–5.0)	6.8±0.2(6.6–7)

##### Description.

**Males.** Body cylindrical and medium sized (881–925 μm in length). Cuticle with transverse rows of dots and a differentiation consisting of two longitudinal rows of distinct larger dots starting posterior the amphidial fovea and extending to the tail tip (2 μm in width). Transverse bars connecting the two larger dots beginning from the middle of the pharynx to the middle of tail. Somatic setae present sparsely along the lateral differentiation in two longitudinal rows, short in the head and tail (9 μm in length), long in the middle of the body (12 μm in length). Inner and outer labial sensilla papilliform. Four cephalic sensilla setiform at the level of amphidial fovea (1.20–1.36 head diameter in length). Head blunt. Amphidial fovea oval (4 µm in width and 3 µm in length), small (26.7%–28.6% corresponding body diameter) and situated 0.47–0.57 head diameter from the anterior end. Buccal cavity cuticularized with a large, hollow and straight dorsal tooth and two small ventrosublateral teeth. Cheilostoma short with longitudinal cuticularized ribs. Pharynx cylindrical, anterior region surrounding buccal cavity slightly swollen, posterior region swollen into an elongated single bulb with plasmatic interruptions resembling a double bulb (18.8–20.0% of pharynx length). Nerve ring slightly posterior to middle pharynx region (53.6–60.9% of pharynx length). Renette cell of secretory-excretory system situated posterior to pharynx bulb, excretory pore located at anterior buccal cavity (6–8 μm from anterior end). Cardia not observed.

The reproductive system monorchid, with extended testis located to the right of intestine. Spicules equal and slightly curved, 23–25 μm (1.0–1.1 cloacal body diameter) along arc, proximal end slightly widened and distal end bifurcated. Gubernaculum jointed without apophysis. Four (1 + 3) precloacal supplements papilliform, anterior three supplements closely distanced and posteriormost supplement distant from the three anterior ones, distance between the supplements 9–12 μm, 8–9 μm, 16–19 μm, respectively, and posteriormost supplement 7–8 μm from cloaca. Tail elongated conical, gradually tapering, 4.5–5.0 cloacal body diameters. Spinneret very short, 1–2 μm in length.

**Females.** Similar to males in most characteristics. Amphidial fovea slit-like (50.0% corresponding body diameter). Cephalic setae short (10–11 μm in length). Reproductive system didelphic, with opposed and reflexed ovaries. Anterior ovary to right of intestine and posterior ovary to left of intestine. Eggs oval shaped, 8–10 × 10–11 μm. Vulva at the middle of the total body. Vagina short.

##### Etymology.

Species epithet *media* refers to the medium body size.

##### Remarks.

*Dichromadoramedia* sp. nov. differs from all other species of the genus *Dichromadora* by the amphidial fovea shape and jointed gubernaculum and it is similar to *D.dissipata*, *D.quadripapillata*, and *D.sinica* in body length and precloacal supplements number. However, it differs from *D.dissipata* in cephalic setae length (10–19 μm vs 9–9.5 μm), spicules length (23–25 μm vs 39 μm), gubernaculum shape (double-jointed without apophysis vs not jointed with dorsal apophysis) and precloacal supplements (1+3 vs 5); differs from *D.quadripapillata* in cephalic setae length (10–19 μm vs 4–5 μm), spicules shape (slightly curved and distally bifurcated vs curved with pointed distal end) and precloacal supplements shape (papilliform vs cup-shaped); differs from *D.sinica* in cuticle differentiation (lateral differentiation with transverse bars vs lateral differentiation without transverse bars), pharynx bulb shape (single bulb with plastic interruptions vs double bulb), spicules shape (slightly curved and distally bifurcated vs distal end with a hook), gubernaculum shape (jointed vs not jointed), and precloacal supplements arrangement (1+3 vs 3+1).

*Dichromadoramedia* sp. nov. shows a close relationship with *D.sinica* in the phylogenetic trees (Figs 5, 6) based on rDNA sequences and it differs by 2% (39 in 1656 bp, including two gaps) in SSU and 5% (38 in 770 bp, including four gaps) in LSU D2–D3 fragment, but they can be morphologically differentiated based on pharynx bulb shape, spicule shape, gubernaculum shape and precloacal supplements.

#### ﻿Genus Neochromadora

Taxon classificationAnimaliaChromadoridaChromadoridae

Micoletzky, 1924

69BD6D41-D91B-555C-80B1-D14D6832AFF8

##### Diagnosis

**(based on Venekey et al. 2019).** Cuticle ornamentation heterogeneous and complex, with lateral differentiation visible as two or three longitudinal rows of large dots. Six small outer labial setae or papillae and four cephalic setae in separate circles. Inner labial sensilla may be conspicuous in one species (*N.munita*). Presence of somatic setae in some species. Amphidial fovea transverse slit-like and loop shaped. Buccal cavity with a dorsal tooth and two ventrosublateral teeth, in some species the dorsal one being larger than the others. Denticles can be present. Pharynx anteriorly not swollen or swollen next to the dorsal tooth. Pharynx with a single well-developed posterior bulb. Male usually with numerous precloacal supplements.

##### Remarks.

The genus *Neochromadora* was erected by Micoletzky (1924) with the type species *Neochromadorapoecilosoma* (de Man, 1893). And six species, *Neochromadoraaberrans* (Cobb, 1930), *Neochromadoracraspedota* (Steiner, 1916), *Neochromadoraedentata* (Cobb, 1914), *Neochromadoraizhorica* (Filipjev, 1929), *Neochromadorapoecilosomoides* (Filipjev, 1918), *Neochromadorasabulicola* (Filipjev, 1918) have been added to *Neochromadora*. Gerlach (1951) described *N.tecta* Gerlach, 1951, redescribed *N.poecilosoma*, *N.izhorica* (Filipjev, 1929), and transferred *Spilipheratrichophora* (Steiner, 1921) to *Neochromadora*. Later Gerlach (1952, 1953b) described *N.attenuate* Gerlach, 1952 and *N.complexa* Gerlach, 1953b. Wieser (1954a) divided *Neochromadora* into two subgenera *Neochromadorina* (Wieser, 1954a) and *Trichodorina* (Wieser, 1954a) based on tooth structure, cervical and somatic setal length, and pharyngeal bulb and described three new species: *N.lateralis* Wieser, 1954a, *N.calathifera* Wieser, 1954a, and *N.torquata* Wieser, 1954a. Later, Wieser (1954b) described another two species *N.amembranata* Wieser, 1954b and *N.brevisetosa* Wieser, 1954b. Afterwards, six species, *N.bonita* Gerlach, 1956, *N.coudenhovei* Wieser, 1956, *N.notocraspedota* Allgén, 1958, *N.appiana* Wieser, 1959, *N.pugilator* Wieser, 1959 and *N.bicoronata* (Wieser, 1959) (synonym *Endeolophosspinosus* (Gerlach, 1957) were described. Wieser (1959) withdrew the subgenus Trichodorina with a redescription of *N.poecilosoma* found in Puget Sound. Later, 13 species (*N.alatocorpa* Hopper, 1961, *N.nitida* Timm, 1961, *N.munita* Lorenzen, 1971, *N.paratecta* Blome, 1974, *N.paramunita* Boucher, 1976, *N.angelica* Riemann, 1976, *N.bilineata* Kito, 1978, *N.oshoroana* Kito, 1981, *N.orientalis* Lemzina, 1982, *N.papillosa* Pastor de Ward, 1985, *N.lineata* Pastor de Ward, 1985, *N.nicolae* Vincx, 1986 and *N.alejandroi* Lo Russo & Pastor de Ward, 2012) were described. Hopper (1963) considered *Neochromadoratrilineata* Schneider, 1943 as incertae sedis due to the unavailability of specimens. Vincx (1986) considered *N.paramunita* as a synonym of *N.munita*. Up to now, 33 species are currently considered as valid (based on Venekey et al. 2019):

1. *Neochromadoraaberrans* (Cobb, 1930) (Antarctic, Commonwealth Bay; = *Spilipheraaberrans* Cobb, 1930)

2. *Neochromadoraalatocorpa* Hopper, 1961 (USA, Alabama)

3. *Neochromadoraalejandroi* Lo Russo & Pastor de Ward, 2012 (Argentina, San Matías gulf)

4. *Neochromadoraamembranata* Wieser, 1954 (Italy, Sampieri)

5. *Neochromadoraangelica* Riemann, 1976b (Germany, Helgoland)

6. *Neochromadoraappiana* Wieser, 1959 (USA, Washington)

7. *Neochromadorabilineata* Kito, 1978 (Japan, Hokkaido)

8. *Neochromadorabonita* Gerlach, 1956 (Brazil, Cananeia)

9. *Neochromadorabrevisetosa* Wieser, 1954 (Italy, Sampieri)

10. *Neochromadoracalathifera* Wieser, 1954b (Chile, Seno Reloncavi)

11. *Neochromadoracomplexa* Gerlach, 1953a (Chile, Seno Ultima Esperanza)

12. *Neochromadoracoudenhovei* Wieser, 1956b (Greece, Piraeus)

13. *Neochromadoracraspedota* (Steiner, 1916) (Arctic Ocean, Barents Sea; = *Chromadoracraspedota* Steiner, 1916)

14. *Neochromadoraedentata* (Cobb, 1914) (Antarctic, Cape Royds; = *Nygmatonchusedentata* (Cobb, 1914) Wieser, 1954, *Spilipheraedentata* Cobb, 1914)

15. *Neochromadoraizhorica* (Filipjev, 1929) (Baltic Sea, Neva Bay; = *Chromadorellaizhorica* Filipjev, 1929)

16. *Neochromadoralateralis* Wieser, 1954 (Chile, Seno Reloncavi)

17. *Neochromadoralineata* Pastor de Ward, 1985a (Argentina, Deseado river)

18. *Neochromadoramunita* Lorenzen, 1972 (Germany, Helgoland; = *Neochromadoraparamunita* Boucher, 1976)

19. *Neochromadoranicolae* Vincx, 1986 (North Sea, Southern Bight)

20. *Neochromadoranitida* Timm, 1961 (Indian Ocean, Bengal Bay)

21. *Neochromadoranotocraspedota* Allgén, 1958 (Uruguay, Uruguay coast)

22. *Neochromadoraorientalis* Lemzina, 1982 (Kyrgyzstan, Lake Issyk-Kul)

23. *Neochromadoraoshoroana* Kito, 1981 (Japan, Oshoro Bay)

24. *Neochromadorapapillosa* Pastor de Ward, 1985 (Argentina, Deseado River)

25. *Neochromadoraparabilineata* sp. nov. (China, Yellow Sea)

26. *Neochromadoraparatecta* Blome, 1974 (Germany, Sylt)

27. *Neochromadorapoecilosoma* (de Man, 1893) (North Sea, English Channel; = *Chromadorapoecilosoma* de Man, 1893)

28. *Neochromadorapoecilosomoides* (Filipjev, 1918) (Black Sea, Kruglaya Bay and Georgievskii Monastery Bay; = *Chromadorapoecilosomoides* Filipjev, 1918)

29. *Neochromadorapugilator* Wieser, 1959 (USA, Washington)

30. *Neochromadorasabulicola* (Filipjev, 1918) (Black Sea, Kruglaya Bay and Georgievskii Monastery Bay; = *Chromadorasabulicola* Filipjev, 1918)

31. *Neochromadoratecta* Gerlach, 1951 (Germany, Amrum Island)

32. *Neochromadoratorquata* Wieser, 1954 (Chile, Seno Reloncavi)

33. *Neochromadoratrichophora* (Steiner, 1921a) (Spain, Canary Islands; = *Spilipheratrichophora* Steiner, 1921, *Neochromadoralongisetosa* Schuurmans-Stekhoven, 1935)

#### ﻿ Neochromadora parabilineata sp. nov.

Taxon classificationAnimaliaChromadoridaChromadoridae

B3AFF3D8-F366-5715-8C27-03CFB7A86B92

https://zoobank.org/27F8BC81-DA38-470D-AD2B-F4B20F302588

**Figure 3.**
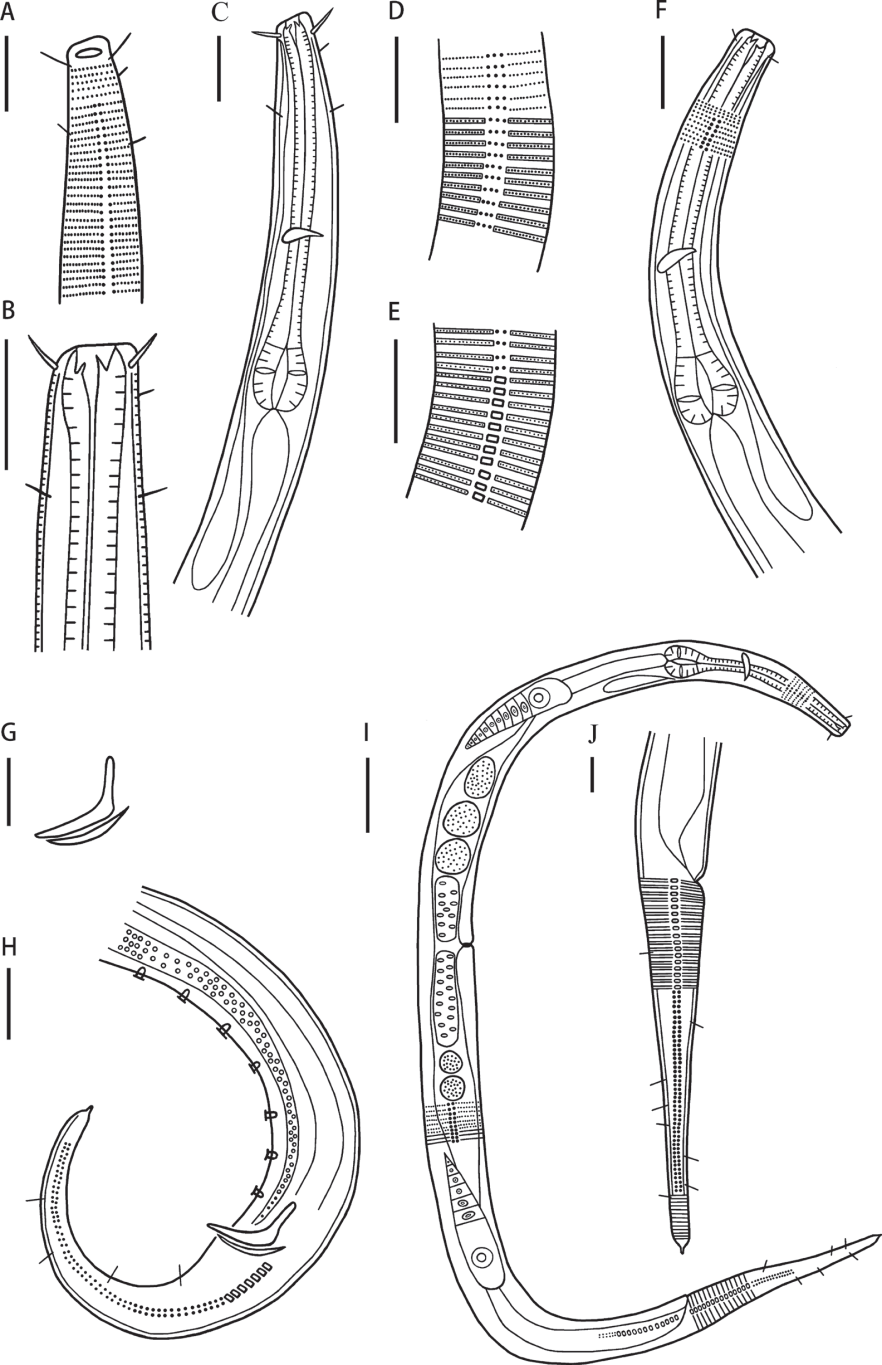
*Neochromadoraparabilineata* sp. nov. **A** lateral view of male anterior region showing cuticle and amphidial fovea (holotype) **B** lateral view of male buccal cavity (holotype) **C** lateral view of male anterior region showing pharyngeal region (holotype) **D** lateral view of male cuticle at pharynx region (holotype) **E** lateral view of male cuticle at middle body (holotype) **F** lateral view of female anterior region showing buccal cavity and pharyngeal region (22HSB11-1-18) **G** lateral view of spicules and gubernaculum (22HSB11-2-20) **H** lateral view of male posterior body, showing precloacal supplements and tail (holotype) **I** lateral view of female whole body (22HSB11-2-18) **J** lateral view of female posterior body showing tail (22HSB11-2-18). Scale bars: 20 µm (**A–H, J**); 50 µm (**I**).

**Figure 4.**
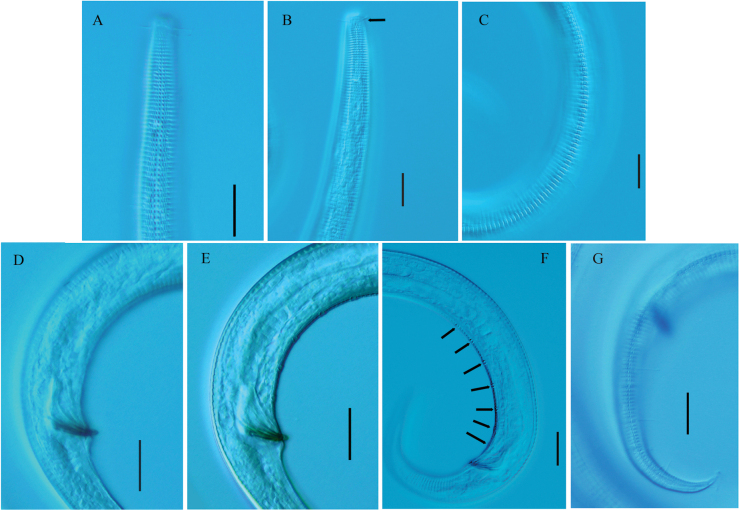
*Neochromadoraparabilineata* sp. nov. **A** lateral view of male anterior region showing cuticle (holotype) **B** lateral view of female anterior region showing amphidial fovea (arrow) (22HSB11-2-18) **C** lateral view of cuticle at middle body (holotype) **D** lateral view of male posterior body, showing spicules (22HSB11-2-20) **E** lateral view of male posterior body, showing gubernaculum (22HSB11-2-20; **F** lateral view of male posterior body, showing precloacal supplements (arrows) (holotype) **G** lateral view of male posterior body, showing tail and cuticle (22HSB11-2-20). Scale bars: 20 µm.

Figs 3 , 4 , Table 4

##### Diagnosis.

Medium body size, buccal cavity with one large hollow dorsal tooth and two small subventral teeth, spicules curved and L-shaped, gubernaculum boat-shaped, seven precloacal supplements cup-shaped, tail conical and gradually tapering.

##### Material examined.

Four males and three females were measured and studied. ***Holotype***: ♂ 1 on slide 22HSB11–2–20; ***paratypes***: ♂ 2 on 22HSB11–1–21, ♂ 3 on 22HSB11–2–18, ♂ 4 on 22HSB11–2–20, ♀ 1 on 22HSB11–2–18, ♀ 2 on 22HSB11–1–18, and ♀ 3 on 22HSB11–2–18.

##### Type locality and habitat.

Rizhao coast, Shandong Province, China, 35°5′N, 119°20′E, 0–2 cm sediment depth, sandy sediment.

##### Measurements.

All measurement data are given in Table 4.

**Table 4.** Measurements of *Neochromadoraparabilineata* sp. nov. (in µm except for ratios).

Characters	Holotype	Paratypes	Paratypes
	male	males (*n* = 3)	females (*n* = 3)
Total body length	878	905.3±39.6(864–943)	913±27.8(881–931)
Maximum body diameter	25	25.7±1.5(24–27)	33.7±5.8(27–37)
Head diameter	12	11.7±1.5(10–13)	11.7±0.6(11–12)
Length of cephalic setae	8	7±0(7–7)	7.3±0.6(7–8)
Buccal cavity depth	10	10.7±3.1(8–14)	7±1(6–8)
Amphidial fovea width	6	6.7±0.6(6–7)	6±0(6–6)
Amphidial fovea from anterior end	3	3±0(3–3)	3±0(3–3)
Body diameter at amphidial fovea	12	11.7±0.6(11–12)	12±0(12–12)
Nerve ring from anterior end	86	80.7±3.8(78–85)	84±6.1(80–91)
Body diameter at nerve ring	23	21.3±0.6(21–22)	23.3±0.6(23–24)
Pharynx length	131	124.7±2.1(123–127)	128±2.6(125–130)
Pharynx bulb length	23	22.7±0.6(22–23)	26±3.6(23–30)
Body diameter at the base of pharynx	24	22.3±1.2(21–23)	26.3±2.9(23–28)
Cloacal/anal body diameter	25	24±0(24–24)	19.3±0.6(19–20)
Spicules length along arc	31	29±1(28–30)	–
Gubernaculum length	21	20.3±1.5(19–22)	–
Vulva from anterior end	–	–	380.3±34.1(341–401)
Body diameter at vulva	–	–	32±4.6(27–36)
Precloacal supplements	7	7	–
V%	–	–	41.6±2.5(38.7–43.1)
Tail length	113	111.3±5.5(105–115)	121.7±12.6(110–135)
a	35.1	35.4±3(32–37.9)	27.6±4.3(25.1–32.6)
b	6.7	7.3±0.3(7–7.6)	7.1±0.1(7–7.2)
c	7.8	8.2±0.7(7.6–9)	7.6±0.8(6.9–8.5)
c’	4.5	4.6±0.2(4.4–4.8)	6.3±0.5(5.8–6.8)

##### Description.

**Males.** Body medium sized (864–943 μm), anterior end truncated and posterior end tapered. Cuticle heterogeneous and complex, five transverse rows of small dots present just posterior to cephalic setae, two or three longitudinal rows of larger dots posterior to the cephalic setae to middle part of body, larger dots changing to rectangular markings from middle body to posterior part of cloaca and rectangular markings changing back to larger dots until tail end. Six inner and six outer labial sensilla papilliform, four setiform cephalic sensilla (0.5–0.7 head diameter in length). Somatic setae present in pharynx and tail region (8 μm in length). Amphidial fovea situated at level of cephalic setae, transverse oval, 6–7 µm in width and 2 µm in length (50–58% corresponding body diameter). Buccal cavity shallow, 10–14 µm in depth. Cheilostoma short with cuticularized longitudinal folds. Pharyngostoma with one large hollow dorsal tooth and two small subventral teeth. Pharynx cylindrical, posterior region swollen into an oval bulb (17.5–17.7% of pharynx length). Nerve ring slightly posterior to middle pharynx region (64.2–66.9% of pharynx length). Secretory-excretory system present; renette cell situated posterior to pharynx bulb, excretory pore at level with cephalic setae. Cardia not observed.

Reproductive system with a single, outstretched testis. Spicules curved and L-shaped, widened at the middle part, 28–31 μm (0.81–0.86 cloacal body diameters) along arc. Gubernaculum short and boat-shaped, distal end tapered. Seven precloacal supplements cup-shaped, distance between the anteriormost and cloaca, the posteriormost and cloaca, 100 μm and 18 μm respectively, distance between supplements almost equal-distanced. Tail conical and gradually tapering, 4.4–4.8 cloacal body diameter in length. Spinneret short, 5 μm in length.

**Females.** Similar to males in most characteristics. Tail slightly longer than in males (5.8–6.8 anal body diameters in length). Reproductive system didelphic, with opposed and reflexed ovaries. Anterior ovary to left of intestine and posterior ovary to right of intestine. Spermatheca present. Vulva situated anterior to middle of body. Vagina short and muscularized.

##### Etymology.

Species epithet *parabilineata* refers to the new species being similar to *Neochromadorabilineata*.

##### Differential diagnosis.

*Neochromadoraparabilineata* sp. nov. is similar to *N.bilineata*, *N.izhorica*, *N.complexa*, and *N.poecilosoma* in precloacal supplements number (7–9). But it differs from *N.bilineata* in body length (864–943 μm vs 567–852 μm), cephalic setae length (7–8 μm vs 4–6 μm), amphidial fovea width (50–58% vs 45% corresponding body diameter), spicules shape and length (L-shaped and widened in the middle portion, 28–31 μm vs arcuate and gradually narrowing, 23–26 μm), and gubernaculum length (19–22 μm vs 15–18 μm); differs from *N.izhorica* in cephalic seta length (7–8 μm vs 14 μm), pharynx shape (posterior bulb obvious vs posterior bulb weak), spicules length (28–31 μm vs 31.5–34.5 μm), gubernaculum shape (distal end tapered vs distal end with anterior-laterally curved tip) (Riemann 1966); differs from *N.complexa* in body length (864–943 μm vs 642 μm), spicules shape (curved and L-shaped with middle portion widened vs L-shaped even in width), gubernaculum shape (boat-shaped vs dorsal part slenderly extended), distance between precloacal supplements (10–15 μm vs 2–5 μm) (calculation based on Gerlach 1953b: fig. 11); differs from *N.poecilosoma* in body length (864–943 μm vs 1900–2000 μm), cephalic setae length (7–8 μm vs 10–14 μm), spicule length (28–31 μm vs 60–65 μm), gubernaculum shape (boat-shaped vs distal tip with small tooth) (de Man 1893).

### ﻿Molecular phylogenetic analysis

**Figure 5.**
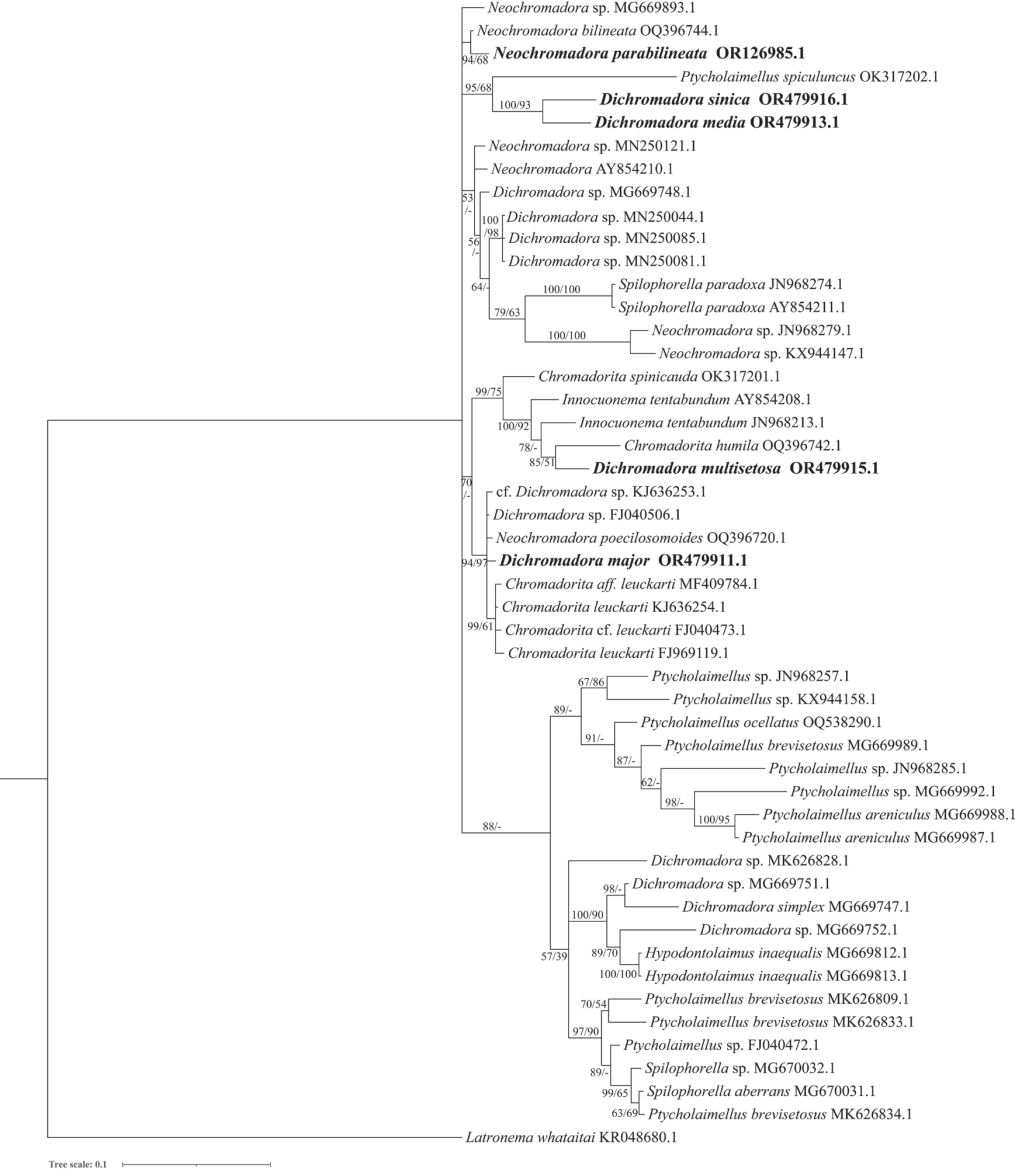
Bayesian inference tree of the subfamily Hypodontolaiminae inferred from Small Subunit (SSU) sequences under the general time-reversible (GTR) + gamma distribution (G) + proportion of invariable sites (I) model. Posterior probability (left) and bootstrap values (right) are given on corresponding clades. The sequences obtained in this study are shown in bold. The scales indicate substitutions per site.

**Figure 6.**
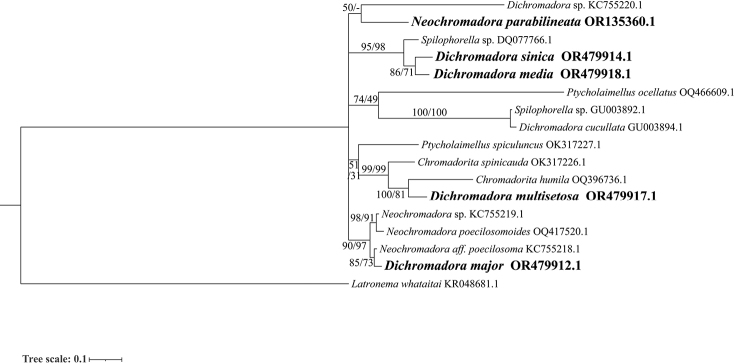
Bayesian inference tree of the subfamily Hypodontolaiminae inferred from the D2-D3 fragment of Large Subunit (LSU) sequences under the general time-reversible (GTR) + gamma distribution (G) model. Posterior probability (left) and bootstrap values (right) are given on corresponding clades. The sequences obtained in this study are shown in bold. The scales indicate substitutions per site.

The ML topology trees which are obtained based on the rDNA gene sequences are mostly in accordance with the BI topology trees, and only the BI trees are shown in Figs 5, 6.

Sequences of seven genera of the subfamily Hypodontolaiminae, *Chromadorita* Filipjev, 1922, *Dichromadora*, *Hypodontolaimus* de Man, 1886, *Innocuonema* Inglis, 1969, *Neochromadora*, *Ptycholaimellus* Cobb, 1920 and *Spilophorella* Filipjev, 1917 are included in the SSU and LSU rDNA analyses. At genus level, only species of *Chromadorita* cluster in one clade (posterior probability 70 in SSU and 99 in LSU, bootstrap value 99 in LSU) but *Chromadorita* is shown as paraphyletic. *Dichromadoramultisetosa* (OR479915), *D.major* (OR479911), *N.poecilosomoides* (OQ396720), and *Innocuonematentabunda* (JN968213, as *Chromadoritatentabunda*) clustered with the *Chromadorita* clade in the SSU analysis. These species share a common character of peribuccal cavity tissue with an asymmetrical dorsal swelling and only one posterior pharynx bulb. However, *Chromadorita* can be morphologically differentiated from them by having the cuticle homogeneous without any lateral differentiation.

Six sequences of genus *Dichromadora* have been identified to species level, but they are in four different clades in both SSU and LSU analyses and therefore paraphyletic. Among the species of *Dichromadora*, *Dichromadoramultisetosa* is the only species with the gubernaculum not being boat-shaped but with dorsal caudal apophysis, and clustered with *Chromadoritahumila* (gubernaculum possessing arched dorsal-caudal apophysis) highly supported by the LSU topology tree (posterior probability 85, bootstrap value 51 in SSU; posterior probability 100, bootstrap value 81 in LSU). *Dichromadorasinica* and *D.media* sp. nov. are highly clustered based on rDNA sequences (posterior probability 100, bootstrap value 93 in SSU; posterior probability 86, bootstrap value 71 in LSU), and can be distinguished based on the pharyngeal bulb, precloacal supplements, and gubernaculum shape.

Species of genus *Neochromadora* differ from other genera of Hypodontolaiminae mainly based on the cuticle ornamentation being heterogeneous and complex. However, the cuticle structure is sometimes seen as a variable character (e.g., Leduc et al. 2017), and sequences of *Neochromadora* present as polyphyletic clades in SSU and LSU topology trees. *Neochromadorabilineata* (OQ396744) and *N.parabilineata* sp. nov. have a close relationship in the SSU analysis (posterior probability 94, bootstrap value 68), but the LSU sequence of *N.bilineata* was missing. Relationships between these two species should be further discussed when more molecular data is available, in combination with morphological characters.

The genera *Ptycholaimellus* and *Spilophorella* are paraphyletic clades in both LSU and SSU analyses. *Ptycholaimellus*, *Hypodontolaimus*, and *Dichromadora* all show morphological similarities with each other and clades are weakly supported in the SSU analysis (posterior probability 88 in SSU). Differences between these three genera are slight, and they are clustered within one morphological group based on buccal cavity, peribuccal pharyngeal tissue, and supplements by Venekey et al. (2019).

## Supplementary Material

XML Treatment for Dichromadora

XML Treatment for Dichromadora media

XML Treatment for Neochromadora

XML Treatment for Neochromadora parabilineata

## ﻿Additional information

### Author ORCIDs

Huixin Liang https://orcid.org/0009-0006-9073-2062

Wen Guo https://orcid.org/0000-0002-4452-0003

Chunming Wang https://orcid.org/0000-0003-3630-0921

### Data availability

All of the data that support the findings of this study are available in the main text.
